# Characterization of Specific Signatures of the Oral Cavity, Sputum, and Ileum Microbiota in Patients With Crohn’s Disease

**DOI:** 10.3389/fcimb.2022.864944

**Published:** 2022-04-13

**Authors:** Kai Xia, Renyuan Gao, Xiaocai Wu, Jing Sun, Jian Wan, Tianqi Wu, Jakub Fichna, Lu Yin, Chunqiu Chen

**Affiliations:** ^1^ Diagnostic and Treatment Center for Refractory Diseases of Abdomen Surgery, Shanghai Tenth People’s Hospital, Tongji University School of Medicine, Shanghai, China; ^2^ Department of Biochemistry, Medical University of Lodz, Lodz, Poland

**Keywords:** Crohn’s disease, microbiota, oral cavity, sputum, ileum, 16S rRNA gene sequence

## Abstract

**Background:**

Crohn’s disease (CD) is a chronic nonspecific inflammatory bowel disease (IBD) with an increasing incidence worldwide. The etiology of CD is still obscure, but microbial dysbiosis has been recognized as an essential factor contributing to CD. However, few studies have revealed the microbiome’s signatures and reciprocal correlations between multiple sites in patients with CD over different disease stages. This study investigated the specific microbial architectures of the oral cavity, sputum, and ileum in patients with CD in the active and remission stages.

**Methods:**

Microbial samples from the oral cavity, sputum, and ileum were collected from patients with CD in the active and remission stages and healthy controls. The microbial composition was assessed by 16S ribosomal RNA (rRNA) gene sequencing. In addition, bioinformatics methods were used to demonstrate the microbial signatures, functional changes, and correlations between microbiota and clinical data in CD.

**Results:**

Compared with healthy controls, a distinct microbiota dysbiosis in the oral cavity, sputum, and ileum of patients with CD was identified, characterized by alterations in microbiota biodiversity and composition. The oral cavity and sputum microbiota showed significantly lower microbial diversity in patients with CD than in healthy controls. In terms of microbiota composition, the microbiota changes in the oral cavity of patients with CD were similar to those in the sputum, while they were different from those in the ileum. In the oral cavity and sputum of patients with CD, a lower relative abundance of *Firmicutes* and *Actinobacteria* was observed compared to healthy controls, which was most prominent in the active stage. In contrast, an increased relative abundance of *Fusobacteria*, *Porphyromonas*, and *Haemophilus* was observed in patients with CD. The predicted metabolic pathways involved in the oral cavity, sputum, and ileum were similar, predominantly involving metabolism, environmental information processing, and genetic information processing.

**Conclusion:**

The results revealed the alterations of microbiota architecture in the oral cavity, sputum, and ileum of patients with CD, which varied across disease stages. Studying microbiota dysbiosis may bring new insights into the etiology of CD and lead to novel treatments.

## Introduction

Crohn’s disease (CD) is a complex chronic inflammatory bowel disease (IBD) that involves genetic susceptibility and environmental triggers (Kong et al., 2021). Although the underlying cause is unknown, a growing number of studies have revealed that intestinal microbial dysbiosis plays an essential role in the occurrence and development of CD due to its impact on intestinal mucosal immunity and barrier function (Bernstein and Forbes, 2017; Sokol et al., 2017; Dovrolis et al., 2020). *Firmicutes* and *Bacteroidetes* synthesize short-chain fatty acids (SCFAs) from carbohydrates, inducing the expression of related proteins required for tight junction formation, contributing to intestinal mechanical barriers (Fesler et al., 2020). In addition, *Bacteroidetes* can produce polysaccharide A (PSA), which has anti-inflammatory effects, inducing Treg cells to release interleukin (IL)-10 to protect the intestinal barrier (Ramakrishna et al., 2019). However, these microbiotas are found in lower numbers in the colon of patients with CD (Lloyd-Price et al., 2019), destroying the intestinal barrier function and increasing CD susceptibility. However, only a few studies have reported the microbiota changes in the ileum and their role in the relapse of CD (Sokol et al., 2020; Olaisen et al., 2021).

CD also has several extraintestinal manifestations, such as glossitis, angular cheilitis, oral mucosal plaques, oral candidiasis, interstitial lung diseases (ILDs), and the like (Katsanos et al., 2015; Danve, 2019). An earlier study showed that some dominant genera in the oral cavity, including *Streptococcus*, *Prevotella*, *Neisseria*, *Haemophilus*, *Veillonella*, and *Gemella*, contributed to dysbiosis in the salivary microbiota of patients with CD (Said et al., 2014). Furthermore, Rautava et al. (2015) demonstrated microbial dysbiosis of the oral cavity in a mouse colitis model. Read et al. (2021) proposed that specific bacteria could translocate from the oral cavity of patients with CD to the intestine and directly cause gut dysbiosis. Similarly, Ubags et al. (Ubags and Marsland, 2017) reported that there was also a potential association between the intestine and the lung *via* intestinal microbiota translocation. However, the microbial signatures from the respiratory tract and the sputum of CD patients remain underestimated.

The present study aimed to explore the characteristics of the oral cavity, sputum, and ileum microbiota of patients with CD in the active and remission stages and investigate the stage-specific microbiota profiles. In addition, the functional alterations of microbiota from multi-ecological sites and correlations between the dominant microbiota strains and clinical factors were also explored.

## Methods

### Patients and Healthy Controls Recruitment

All the study participants were enrolled from the Shanghai Tenth People’s Hospital from July 2021 to August 2021, including patients in the active stage, remission stage, and healthy controls. The diagnosis of CD was based on a comprehensive evaluation of clinical symptoms, laboratory examinations, endoscopic features, radiological findings, and histological features by two experienced physicians (Ding et al., 2020). Patients with CD in the active stage all underwent ileostomy, and the patients in the remission stage came to the hospital for the reversal of ileostoma. The exclusion criteria included severe oral and respiratory diseases diagnosed within 1 month, such as dental caries, periodontitis, oral malignant tumor, oral CD, peri-oral CD, pneumonia, and lung cancer. Meanwhile, a history of probiotics or prebiotic consumption and antibiotic use within 1 month and other acute or chronic gastrointestinal disorders were also excluded. People who came to the hospital for physical examination were recruited as healthy controls. Among them, people with a history of chronic inflammatory conditions including IBD and current gastrointestinal or respiratory symptoms were excluded. The Ethics Committee of Shanghai Tenth People’s Hospital approved this study protocol (Ethical Approval Number: SHSY-IEC-4.1/21-145/01). Informed consent was obtained from all patients. All procedures were performed following the Declaration of Helsinki and its later amendments. This study has been registered in ClinicalTrial.gov (NCT04965584).

### Samples Collection and Clinical Data

The nutrition risk screening score (2002) (Kondrup et al., 2003) and Crohn’s disease activity index (CDAI) (Yoshida, 1999) were recorded for each recruited patient. A CDAI score over 150 was regarded as the active stage, whereas a CDAI score of 150 or below was defined as the remission stage.

**Figure 1:**
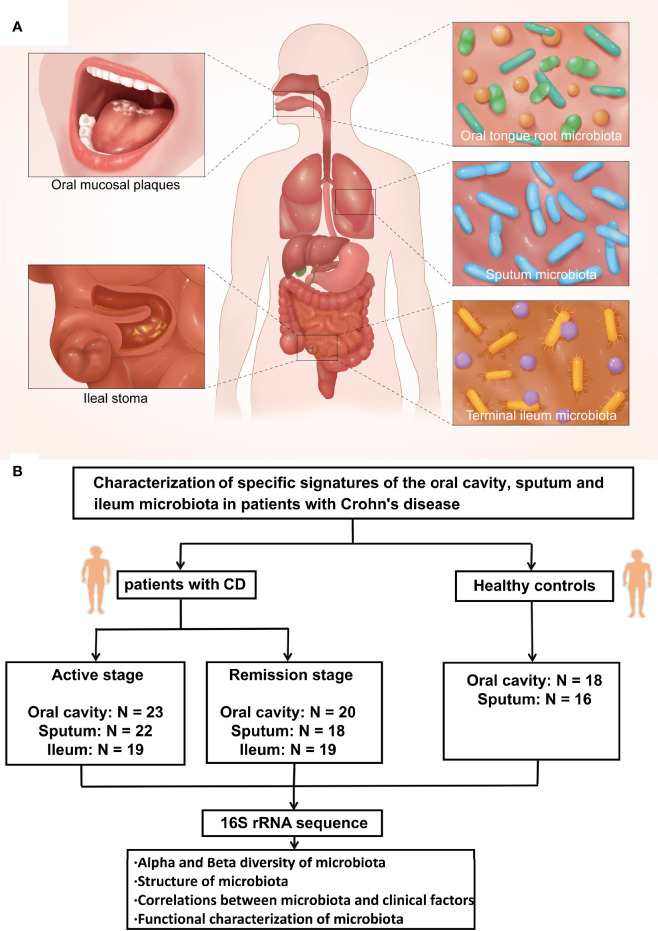
Human body model diagram and experimental flow diagram. **(A)** Microbial samples were collected from the tongue base, sputum and ileal stoma. **(B)** Microbial samples from the oral cavity, sputum, and ileum were collected from patients with CD in the active and remission stages and healthy controls. The microbial composition was assessed by 16S rRNA gene sequencing. Bioinformatics methods were used to demonstrate the alpha and beta diversity of microbiota, the structure of microbiota, correlations between microbiota and clinical factors, and functional characterization of microbiota.

Saliva samples from the tongue base of the recruited patients and healthy controls were collected with a sterile cotton swab and immediately placed into a 2-ml sterile container. Before sputum sample collection, the participants were instructed to gargle three times and inhale 5 ml hypertonic sodium chloride solution by atomization for 10 min, then coughed hard to obtain sputum samples. Fecal samples from the ileal stoma were collected with a sterile cotton swab and placed into a 2-ml sterile container. In addition, 2 g of cefmetazole was routinely used intraoperatively, and there was no history of antibiotic use before surgery for those patients (**Figure 1A**). All samples were transported to the laboratory with an ice pack within 2 h, frozen immediately, and stored at −80°C before analyses.

### DNA Extraction and PCR Amplification

Microbial DNA was extracted from all samples using the E.Z.N.A.^®^ soil DNA Kit (Omega Bio-tek, Norcross, GA, USA) according to the manufacturer’s instructions. The final DNA concentration and purification were determined by a NanoDrop 2000 UV-vis spectrophotometer (Thermo Scientific, Wilmington, USA), and the DNA quality was checked by 1% agarose gel electrophoresis. The V3–V4 hypervariable regions of the bacteria 16S ribosomal RNA (rRNA) gene were amplified with primers 338F (5′- ACTCCTACGGGAGGCAGCAG-3′) and 806R (5′-GGACTACHVGGGTWTCTAAT-3′) with the thermocycler PCR system (GeneAmp 9700, ABI, USA). The PCR reactions were conducted using the following procedure: 3 min of denaturation at 95°C, 27 cycles of 30 s each at 95°C, 30 s for annealing at 55°C, and 45 s for elongation at 72°C, and a final extension at 72°C for 10 min. PCR reactions were performed in triplicate, with 20 μl mixture containing 4 μl of 5× FastPfu buffer, 2 μl of 2.5 mM deoxyribonucleotide triphosphates (dNTPs), 0.8 μl of each primer (5 μM), 0.4 μl of FastPfu polymerase, and 10 ng of template DNA. The resulting PCR products were extracted from a 2% agarose gel, further purified using the AxyPrep DNA Gel Extraction Kit (Axygen Biosciences, Union City, CA, USA) and quantified using QuantiFluor™-ST (Promega, USA) according to the manufacturer’s protocol.

### Illumina MiSeq Sequencing and Processing of Sequencing Data

Purified amplicons were pooled in equimolar ratios and paired-end sequenced (2 × 300) on an Illumina MiSeq platform (Illumina, San Diego, USA) according to the standard protocols by Majorbio Bio-Pharm Technology Co. Ltd. (Shanghai, China).

Raw fastq files were demultiplexed, quality filtered by Trimmomatic, and merged by FLASH with the following criteria: (i) the reads were truncated at any site receiving an average quality score <20 over a 50-bp sliding window; (ii) primers were exactly matched, allowing two nucleotide mismatching, and reads containing ambiguous bases were removed; and (iii) sequences whose overlap was longer than 10 bp were merged according to their overlap sequence.

Operational taxonomic units (OTUs) were clustered with 97% similarity cutoff using UPARSE (version 7.1 http://drive5.com/uparse/), and chimeric sequences were identified and removed using UCHIME. The taxonomy of each 16S rRNA gene sequence was analyzed by the RDP Classifier algorithm (http://rdp.cme.msu.edu/) against the Silva (SSU123) 16S rRNA database using a confidence threshold of 70%.

### Statistical Analysis

Alpha diversity was analyzed using the Mothur software (version 1.30.2 https://www.mothur.org/wiki/Download_mothur). To evaluate alpha diversity, the Chao index was calculated, representing the abundance of microbiota, while the Shannon index represented microbiota diversity. A Venn diagram was drawn by Venn Diagram of software R (version 3.3.1) to show the similarity and overlap of microbiota composition of each group. In addition, the Community Bar diagram was drawn by software R (version 3.3.1) to show the abundance of microbiota in multi-ecological sites. The principal coordinates analysis (PCoA) is a multivariate statistical analysis method that reduces the dimensionality of multidimensional data to extract the most important elements. The differences between groups are presented in a two-dimensional coordinate graph (Liu et al., 2020). Furthermore, the Wilcoxon rank-sum test (R package “stats” and python package “scipy”) was used to analyze the inter-group differences of microbiota. A correlation heatmap was drawn with the “pheatmap” package of software R (version 3.3.1), visually reflecting the association between clinical factors and dominant microbiotas in the corresponding ecological sites. In addition, the PICRUSt software (version 1.1.0 http://picrust.github.io/picrust/) was used to predict the function of 16S in the amplicon-sequencing results. The Benjamini–Hochberg method was used to correct multiple comparisons, and an false discovery rate (FDR) value <0.05 was considered statistical significant (Gao et al., 2021). p < 0.05 at two sides was considered significant in all statistical analyses **Figure 1B**.

### Data Access

The 16S rRNA gene sequence data sets have been deposited in the NCBI Sequence Read Archive (SRA) under the study accession number PRJNA773617.

## Results

### Baseline Demographic and Clinical Characteristics of Patients With CD and Healthy Controls

A total of 43 patients (23 patients in the active stage and 20 patients in the remission stage) and 18 healthy controls were recruited in this study (**Table 1**). Patients with CD were at higher nutritional risk (p < 0.001) than their healthy counterparts. Most patients with CD had a regular diet and had no history of smoking, alcohol, or hematochezia, similar to the healthy controls. Moreover, the CD lesions were mainly located in the ileocolon. The levels of hematological indexes, including C-reactive protein (CRP), white blood cell (WBC), hemoglobin, albumin, prealbumin, and erythrocyte sedimentation rate (ESR), were significantly different between the active and remission stages (all p < 0.05). However, the levels of inflammatory biomarkers, including procalcitonin (PCT), IL-1, IL-6, IL-8, and IL-10, were similar between the active and remission stages of patients with CD (**Table 1**).

**Table 1:** Baseline demographic and clinical characteristics of patients with CD and healthy controls.

Characteristics	Active stage (N=23)	Remission stage (N=20)	Healthy controls (N=18)	p-value
Age	33.22±13.94	36.90±10.76	28.72±7.90	.096
Gender (male/female)	17/6	14/6	15/3	.701
BMI (kg/m^2^)	19.40±4.38	20.32±3.69	22.85±3.30	.020
CDAI score	289.58±74.88	78.45±26.46	N/A	<.001
Lesion location*				
L1/L2/L3/L4	7/0/16/0	3/0/17/0	N/A	.405
Duration of disease (years)	4.12±5.21	4.80±5.37	N/A	.669
NRS score (2002)	2.91±1.00	3.55±1.43	0.39±1.20	<.001
Diet				
Regular diet/enteral nutrition/mixed diet	17/6/2	13/2/5	18/0/0	.011
Smoke				
Never/past/current	15/3/5	15/5/0	15/1/2	.116
Alcohol				
Never/past/current	14/4/5	12/3/5	11/0/7	.374
Bristol stool scale^#^				
Type 1/2/3/4/5/6/7	0/2/1/6/5/8/1	0/1/2/1/3/12/1	1/0/4/13/0/0/0	<.001
Hematochezia (Yes/No)	7/16	0/20	0/18	.001
Hematological index				
CRP (mg/L)	49.82±57.39	3.63±1.71	N/A	.001
WBC (*10^9^/L)	7.28±3.71	5.41±1.30	N/A	.031
Hemoglobin (g/L)	115.48±18.73	129.35±11.97	N/A	.007
Albumin (g/L)	35.03±6.00	41.68±3.33	N/A	<.001
Prealbumin (mg/L)	146.48±68.77	273.15±71.26	N/A	<.001
ESR (mm/h)	33.09±18.79	20.00±15.21	N/A	.017
PCT (ng/ml)	0.50±1.57	0.05±0.01	N/A	.184
IL-1 (pg/ml)	6.90±6.56	6.91±4.38	N/A	.996
IL-6 (pg/ml)	35.39±100.30	5.73±4.58	N/A	.171
IL-8 (pg/ml)	101.51±107.93	86.93±99.05	N/A	.649
IL-10 (pg/ml)	8.23±11.76	5.00±0	N/A	.202
Medicine				
**Mesalazine/Azathioprine/Adamuzumab/Remicade/Steroids/Others**	11/5/7/6/2/2	1/6/5/12/0/1	N/A	.018

BMI, body mass index; CDAI score, Crohn’s disease activity index score; NRS score (2002), nutrition risk screening score (2002); CRP, C-reactive protein; WBC, white blood cell; ESR, erythrocyte sedimentation rate; PCT, procalcitonin; IL-1, interleukin-1; IL-6, interleukin-6; IL-8, interleukin-8; IL-10, interleukin-10; N/A, not applicable.

Lesion location* L1: terminal ileum; L2: colon; L3: ileocolon; L4: upper digestive tract.

Bristol stool scale^#^ Type 1: separate hard lumps, like nuts; type 2: sausage-shaped but lumpy; type 3: like a sausage but with cracks on its surface; type 4: like a sausage or snake, smooth and soft; type 5: soft blobs with clear-cut edges; type 6: fluffy pieces with ragged edges, a mushy stool; type 7: watery, no solid pieces.

### Alpha and Beta Diversity of Microbiota in Patients With CD and Healthy Controls

**Figure 2:**
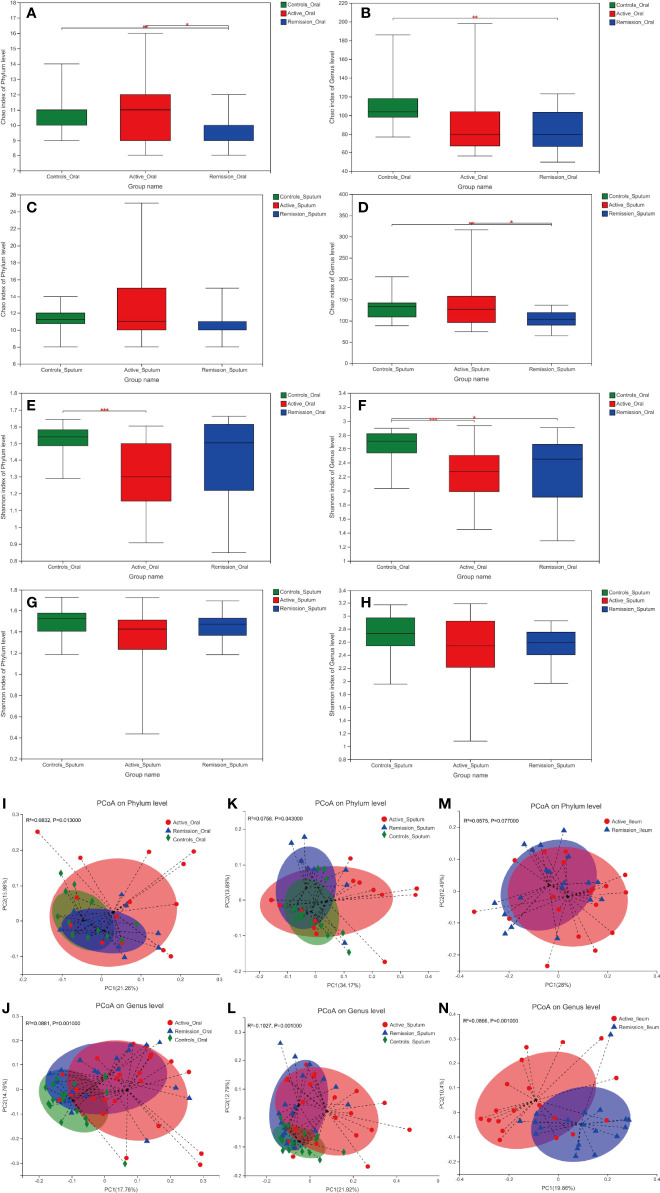
Alpha and beta diversity of microbiota in the oral cavity and sputum of patients with CD and healthy controls. **(A–D)** Chao diversity index of phylum and genus levels in the samples of oral cavity and sputum. **(E–H)** Shannon diversity index of phylum and genus levels in the samples of oral cavity and sputum. **(I–N)** Principle coordinate analysis (PCoA) on the phylum and genus levels using the unweighted UniFrac distance and Adonis test. Red color represents samples from active stage, blue indicates remission stage, while green color indicates healthy controls. *, **, and *** correspond to p-values <0.05, 0.01, and 0.001, respectively.

In this study, the Chao and Shannon indexes were used to analyze the alpha diversity of microbiota. The Chao index was used to reflect the abundance of microbiota in the samples, and the Shannon index was used to indicate microbiota diversity. Patients in the remission stage had a significantly lower Chao index of oral microbiota than healthy controls on both phylum and genus levels (p = 0.006 and p = 0.002) (**Figures 2A, B**). Similar results were observed in sputum samples (**Figures 2C, D**). In addition, the Shannon index of the oral microbiota in healthy controls was significantly higher than that in both groups of patients with CD (**Figures 2E, F**). However, there was no significant difference in sputum microbiota in patients with CD and healthy controls (**Figures 2G, H**). As for the ileal microbiota, the Chao and Shannon indexes of the active stage were higher than those of the remission stage, but the differences were not statistically significant (**Supplementary Figures S1A–D**).

Principle coordinate analysis (PCoA) using the unweighted UniFrac distances also demonstrated that the microbiota in the oral cavity (p = 0.013 and p = 0.001) and sputum (p = 0.043 and p = 0.001) microbiota differed significantly among different stages of CD and healthy controls on phylum and genus levels, respectively (**Figures 2I–L**). In terms of ileal microbiota, a notable difference was observed between active and remission stages on the genus level (p = 0.001) but not on the phylum level (**Figures 2M, N**).

### Structure Analysis of the Oral Microbiota in Patients With CD

**Figure 3:**
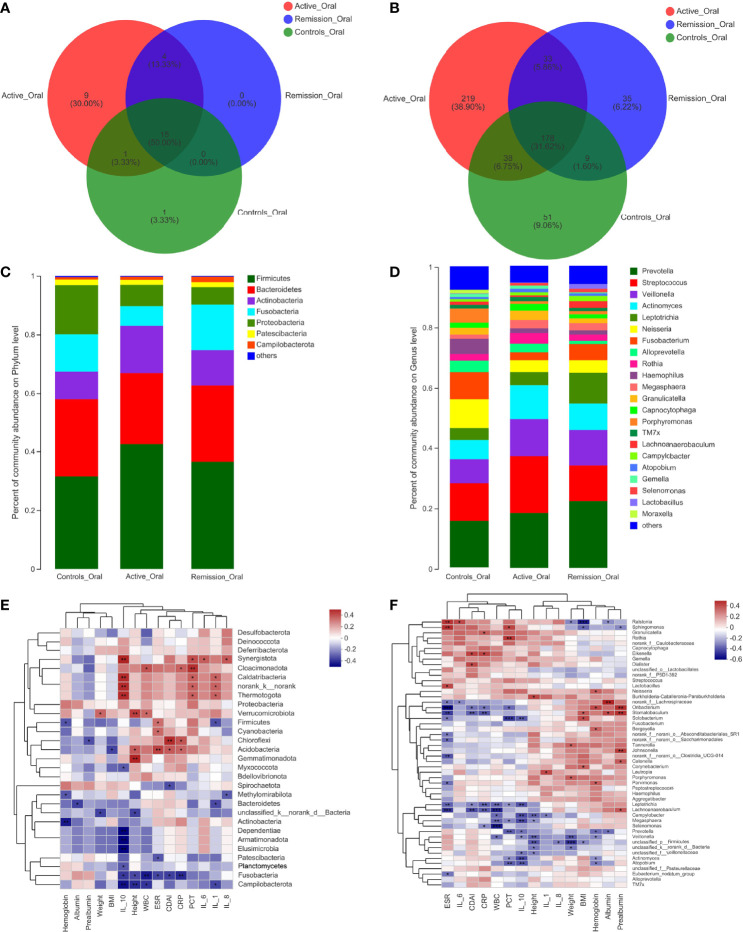
Structure analysis of the oral microbiota in patients with CD. **(A, B)** Venn diagram indicating the overlap of OTUs in the categories on the phylum and genus levels, respectively. **(C, D)** Barplot of the relative abundances of different taxa on the phylum and genus levels, respectively. **(E, F)** Heatmap revealing the association between oral microbiota and clinical factors on the phylum and genus levels, respectively. *, **, and *** correspond to p-values <0.05, 0.01, and 0.001, respectively.

The total number of OTUs across all samples was 65 on the phylum level, among which 29 were identified in the active stage, 19 in the remission stage, and 17 in the healthy controls. Fifteen of these OTUs were common to all three groups (**Figure 3A**). The relative abundances of *Firmicutes* and *Actinobacteria* were the highest in the active stage and were lower in the remission stage of CD and lowest in the healthy controls. On the contrary, *Bacteroidetes* and *Fusobacteria* were predominant in the remission stage of CD and healthy controls (**Figure 3C**).

**Figure 4:**
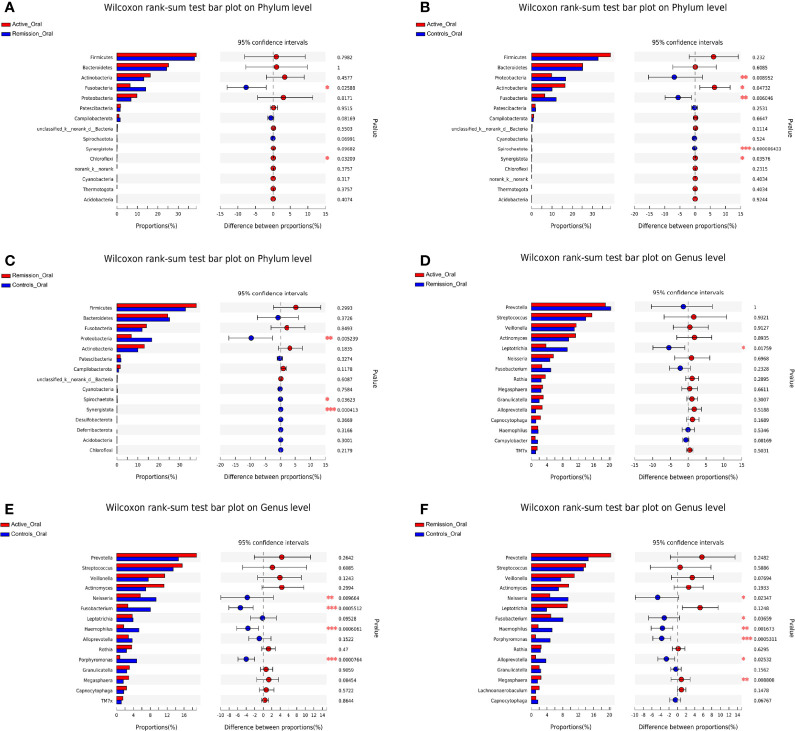
Inter-group difference analysis of microbiota in the oral cavity of patients with CD and healthy controls. **(A–C)** Wilcoxon rank-sum test bar plot of different taxa on the phylum level. **(D–F)** Wilcoxon rank-sum test bar plot of different taxa on the genus level. *, **, and *** correspond to p-values <0.05, 0.01, and 0.001, respectively.

We then aimed to investigate the stage-specific oral microbial profiles. Regarding the oral microbiota, *Fusobacteria* was more abundant in the remission stage of CD (p = 0.026), while *Chloroflexi* was more abundant in the active stage on the phylum level (p = 0.032) (**Figure 4A**). The relative abundances of *Actinobacteria* and *Synergistota* were higher in the active stage of CD (p = 0.047 and p = 0.036), while *Fusobacteria* and *Spirochaetota* were more abundant in the healthy controls (p = 0.006 and p < 0.001) (**Figure 4B**). In addition, the relative abundances of *Spirochaetota* and *Synergistota* were higher in the healthy controls than in the remission stage of CD (p = 0.036 and p < 0.001, respectively) (**Figure 4C**).

On the genus level, 999 OTUs were identified in total, 468 were identified in the active stage, 255 in the remission stage, and 276 in the healthy controls. A total of 178 OTUs were shared across all three groups. The active stage group and the remission stage group had 33 overlapping OTUs, while the active stage group and the control group shared 38 OTUs. In contrast, the remission stage group and the control group had nine OTUs in common (**Figure 3B**). The relative abundances of *Veillonella*, *Actinomyces*, *Megasphaera*, and *Atopobium* decreased gradually from the active stage and remission stage of CD to healthy controls. However, the relative abundances of *Fusobacterium*, *Haemophilus*, and *Porphyromonas* increased gradually along the same sequence. Furthermore, *Campylobacter* was abundant in the oral cavity of patients with CD but less predominant in the healthy controls (**Figure 3D**). The only oral microbiota with a significant difference between the active and remission stages was *Leptotrichia* (p = 0.018), which was more abundant in the remission stage (**Figure 4D**). The relative abundances of *Fusobacterium*, *Haemophilus*, *Porphyromonas*, and *Neisseria* demonstrated a higher prominence in healthy controls than in the active stage of CD (p < 0.001, p < 0.001, p < 0.001, and p = 0.010) (**Figure 4E**). Comparing the remission stage to healthy controls, *Porphyromonas*, *Haemophilus*, *Neisseria*, *Fusobacterium*, and *Alloprevotella* were more abundant in the healthy controls, whereas *Megasphaera* was more abundant in the remission stage. These differences in microbiotas were statistically significant (all p < 0.05) (**Figure 4F**).

### Structure Analysis of the Microbiota of Sputum in Patients With CD

**Figure 5:**
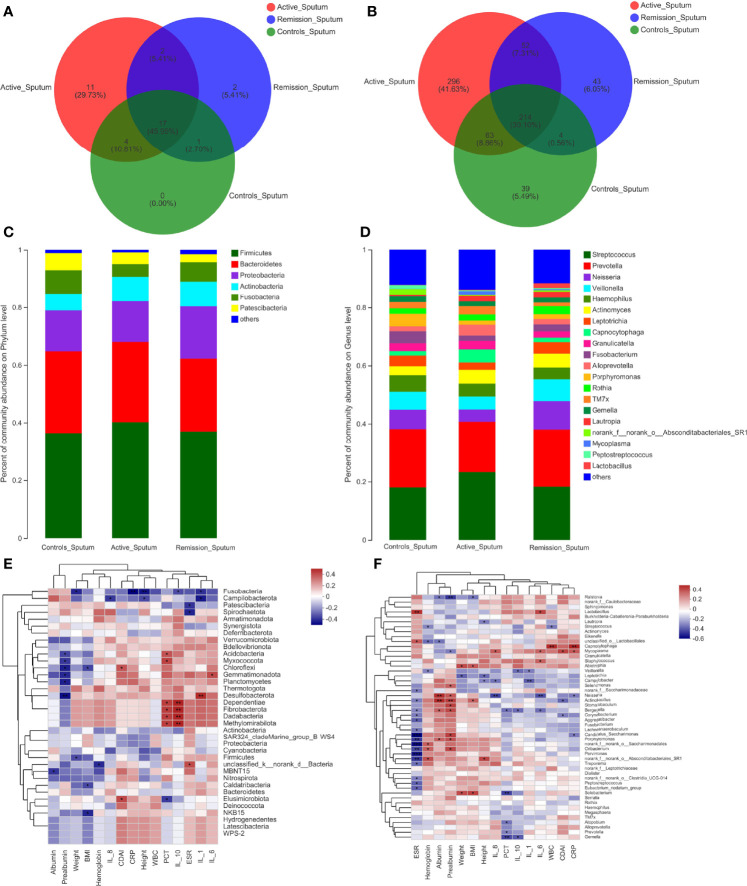
Structure analysis of the sputum microbiota in patients with CD. **(A, B)** Venn diagram indicating the overlap of OTUs in the categories on the phylum and genus levels, respectively. **(C, D)** Barplot of the relative abundances of different taxa on the phylum and genus levels, respectively. **(E, F)** Heatmap revealing the association between sputum microbiota and clinical factors on the phylum and genus levels, respectively. *, **, and *** correspond to p-values <0.05, 0.01, and 0.001, respectively.

The total number of OTUs across all samples was 78 on the phylum level; 34 were identified in the active stage, 22 in the remission stage, and 22 in the healthy controls, respectively. Seventeen OTUs were common to all three groups (**Figure 5A**). The relative abundances of *Firmicutes* and *Actinobacteria* were highest in the active stage of CD and lowest in healthy controls, while the contrary was observed for *Fusobacteria* (**Figure 5C**).

**Figure 6:**
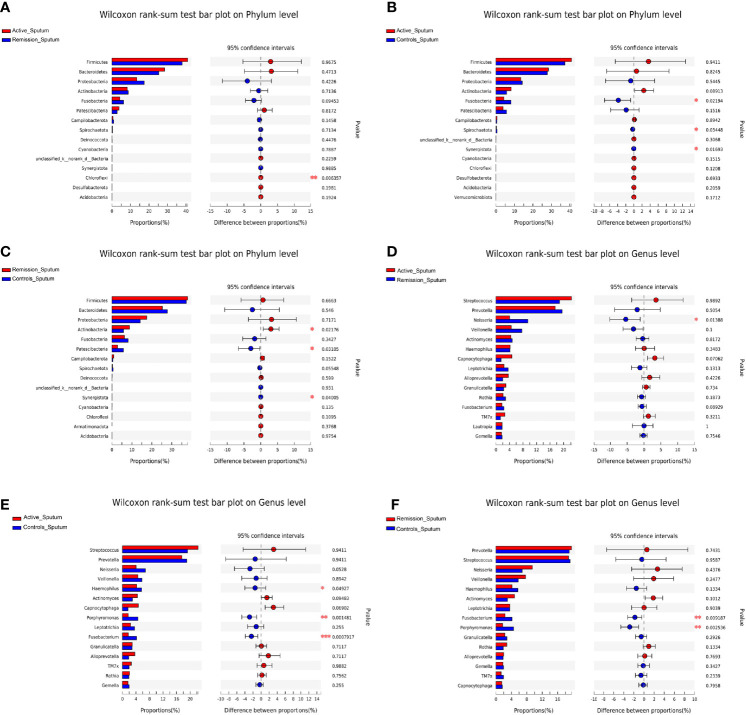
Inter-group difference analysis of sputum microbiota of patients with CD and healthy controls. **(A–C)** Wilcoxon rank-sum test bar plot of different taxa on the phylum level. **(D–F)** Wilcoxon rank-sum test bar plot of different taxa on the genus level. *, **, and *** correspond to p-values <0.05, 0.01, and 0.001, respectively.

Regarding the sputum microbiota, *Chloroflexi* was the only microbiota that was significantly enriched in the active stage (p = 0.006) (**Figure 6A**). The relative abundances of *Fusobacteria*, *Spirochaetota*, and *Synergistota* were significantly higher in the healthy controls, especially compared to the active stage of CD (p = 0.022, p = 0.034, and p = 0.017, respectively) (**Figure 6B**). Furthermore, *Actinobacteria* was more abundant in the remission stage of CD (p = 0.022), whereas *Patescibacteria* and *Synergistota* were more abundant in the healthy controls (p = 0.031 and p = 0.040) (**Figure 6C**).

On the genus level, the total number of OTUs across all samples was 1,258. The three groups shared 214 OTUs, active stage and remission stage groups shared 52 OTUs, the active stage and control groups shared 63 OTUs, and the remission stage and control groups shared 4 OTUs (**Figure 5B**). The relative abundance of *Streptococcus* declined gradually from the active stage, remission stage of CD to healthy controls. On the contrary, the relative abundances of *Prevotella*, *Fusobacterium*, *Porphyromonas*, and *Haemophilus* were lowest in the active stage of CD and highest in the healthy controls (**Figure 5D**). The only sputum microbiota with a significant difference between the active and remission stages was *Neisseria* (p = 0.014), as it was more abundant in the remission stage (**Figure 6D**). In contrast, the relative abundances of *Fusobacterium*, *Haemophilus*, and *Porphyromonas* were much higher in the healthy controls (p < 0.001, p = 0.049, and p = 0.001) (**Figure 6E**). Furthermore, the relative abundances of *Fusobacterium* and *Porphyromonas* were significantly higher in the healthy controls than in the remission stage group (p = 0.009 and p = 0.004) (**Figure 6F**).

### Structure Analysis of the Ileal Microbiota in Patients With CD

**Figure 7:**
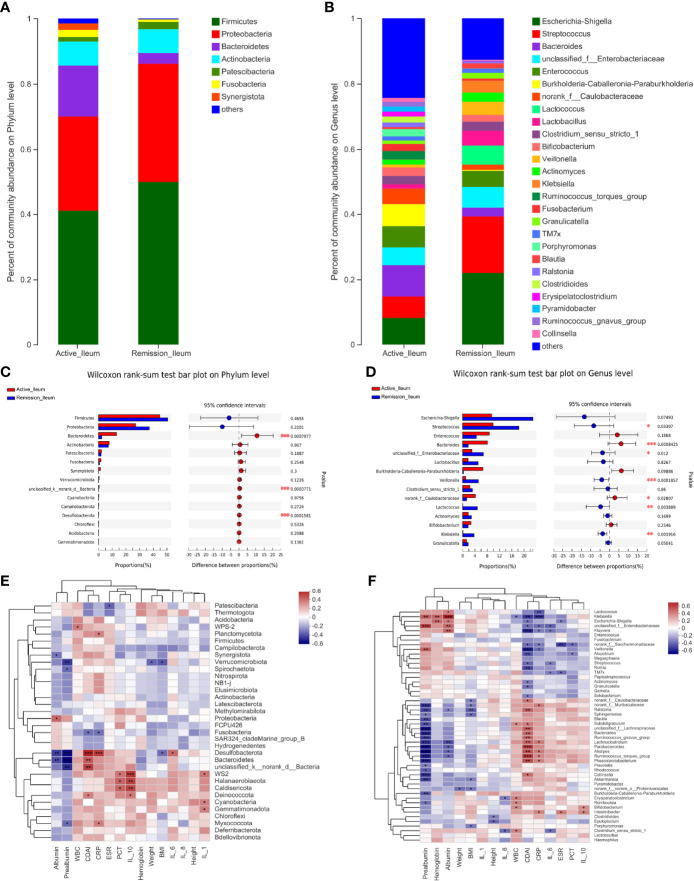
Structure analysis of the ileal microbiota in patients with CD. **(A, B)** Barplot of the relative abundances of different taxa on the phylum and genus levels, respectively. **(C, D)** Wilcoxon rank-sum test bar plot of different taxa on the phylum and genus levels, respectively. **(E, F)** Heatmap revealing the association between ileal microbiota and clinical factors on the phylum and genus levels, respectively. *, **, and *** correspond to p-values <0.05, 0.01, and 0.001, respectively.

The total number of OTUs across all samples was 56 on the phylum level, 30 were identified in the active stage, and 26 in the remission stage. Twenty-two OTUs were found in common in the active stage and remission stage groups (**Supplementary Figure S1E**). The amount of OTUs on the genus level was 1,105, and the two groups shared 353 OTUs. 321 were exclusively identified in the active stage and 78 in the remission stage (**Supplementary Figure S1F**). The relative abundances of *Bacteroidetes* and *Desulfobacterota* were much higher in the active stage on the phylum level (all p < 0.001) (**Figure 7C**). However, the relative abundances of *Firmicutes* and *Patescibacteria* were higher in the active stage than in the remission stage of CD, but the differences between these microbiotas were not statistically significant (**Figures 7A, C**). On the genus level, the relative abundance of *Bacteroides* was much higher in the active stage of CD (p < 0.001). On the contrary, the relative abundances of *Streptococcus*, *Veillonella*, and *Lactococcus* were higher in the remission stage of CD (p = 0.033, p < 0.001, and p = 0.004) (**Figures 7B, D**).

### Correlations Between Dominant Microbiota and Clinical Factors in Patients With CD

To identify the correlations between dominant microbiota and several clinical factors, the Spearman’s rank correlation coefficient was used. Regarding the oral microbiota, ESR levels (R = 0.308, p = 0.045) were positively associated with *Firmicutes*, whereas IL-1 (R = −0.324, p = 0.034) and hemoglobin levels (R = −0.317, p = 0.038) in the blood of patients with CD showed negative correlations (**Figure 3E**). Similarly, the relative abundance of *Firmicutes* in the sputum samples was also positively correlated with ESR levels (R = 0.343, p = 0.030), while it was negatively correlated with hemoglobin levels (R = −0.375, p = 0.017) (**Figure 5E**). However, no significant correlations between the relative abundance of *Firmicutes* and clinical factors were observed in the ileal samples (**Figure 7E**). On the genus level, PCT levels in the blood of patients with CD were found to be negatively correlated with the relative abundance of *Prevotella* in the oral and sputum samples (R = −0.407, p = 0.007 and R = −0.321, p = 0.043, respectively) (**Figures 3F**, **5F**). Moreover, IL-10 (R = −0.360, p = 0.018), hemoglobin (R = −0.377, p = 0.013), and albumin (R = −0.369, p = 0.015) concentrations were also negatively correlated with the relative abundance of *Prevotella* in the oral samples (**Figure 3F**). The relative abundance of *Streptococcus* in the sputum samples was negatively correlated with white blood cell count (R = −0.334, p = 0.035) and hemoglobin concentration (R = −0.313, p = 0.049), whereas no significant correlations in the oral samples were observed (**Figures 3F**, **5F**). Regarding the ileal microbiota, hemoglobin (R = 0.414, p = 0.010) and albumin (R = 0.367, p = 0.023) concentrations showed positive correlations with the relative abundance of *Escherichia–Shigella*, whereas CDAI score (R = −0.353, p = 0.030) and ESR levels (R = −0.333, p = 0.041) in the blood were negatively associated with the microbiota. In addition, CDAI score (R = −0.390, p = 0.016) and IL-6 concentration (R = −0.335, p = 0.040) were negatively associated with the relative abundance of *Streptococcus* (**Figure 7F**).

### Functional Characterization Along With Altered Microbiota Composition in Patients With CD

**Figure 8:**
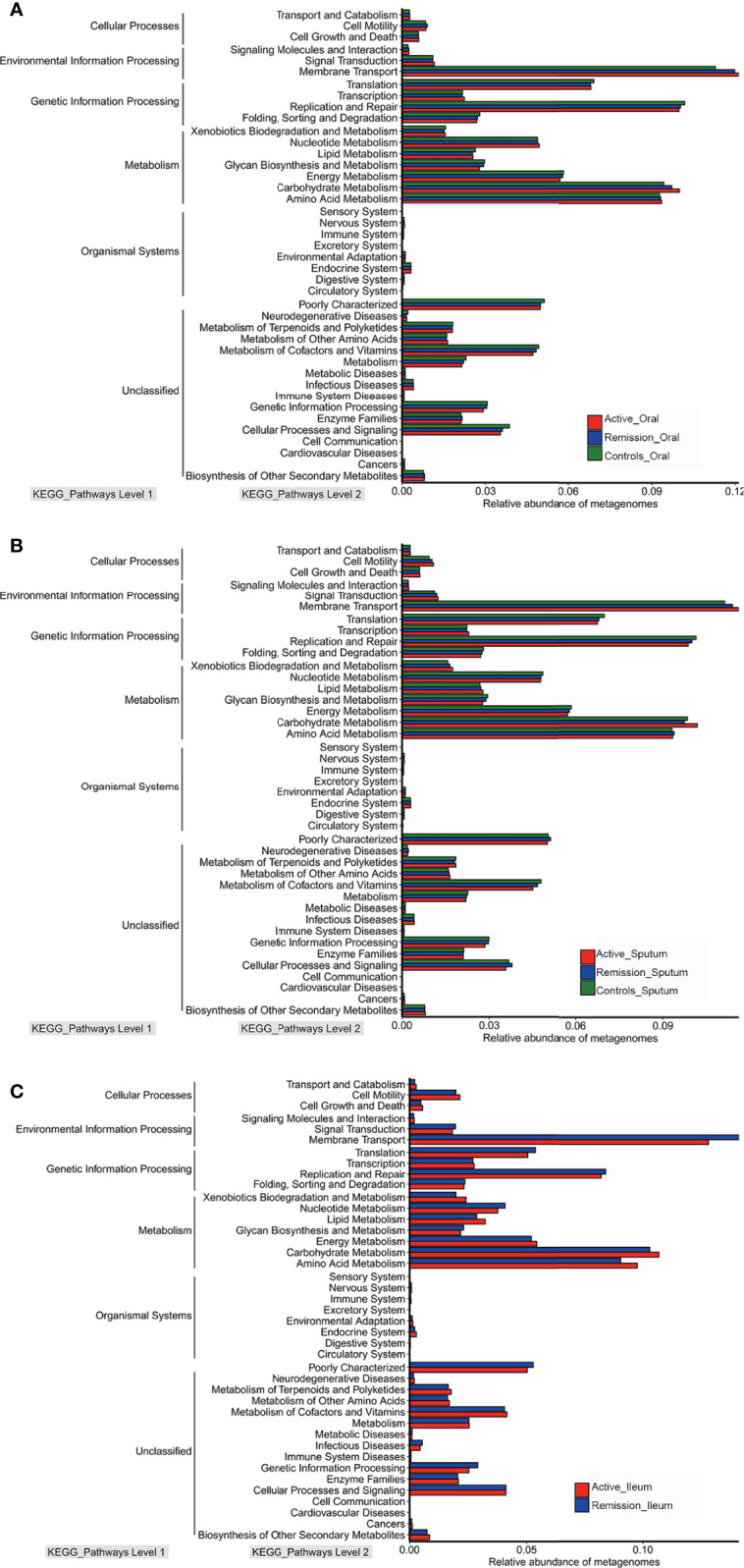
Relative abundances of metagenomes of different KEGG pathways level in microbiota of patients with CD. **(A)** Relative abundances of metagenomes of different KEGG pathways level in oral microbiota of patients with CD. **(B)** Relative abundances of metagenomes of different KEGG pathways level in sputum microbiota of patients with CD. **(C)** Relative abundances of metagenomes of different KEGG pathways level in ileal microbiota of patients with CD. Red color represents samples from active stage, blue indicates remission stage, while green color indicates healthy controls, respectively.

To characterize the microbial functions alterations in patients with CD, we analyzed clusters of orthologous group (COG) functional classifications for microbiota and characterization of different metagenomes of Kyoto Encyclopedia of Genes and Genomes (KEGG) pathways levels. Oral microbial gene processes, such as translation, ribosomal structure and biogenesis, general function prediction only, amino acid transport and metabolism, replication, recombination and repair, and cell wall/membrane/envelope biogenesis pathways were highly abundant in all samples (**Supplementary Figure S2A**). In addition, the microbiota of sputum samples showed similar functional characteristics (**Supplementary Figure S2B**). However, the most active COG functional categories in the ileum were amino acid transport and metabolism, followed by general function prediction only, carbohydrate transport, and metabolism and transcription (**Supplementary Figure S2C**). Regarding the relative abundance of different metagenomes of KEGG pathways levels, the predicted metabolic pathways in the oral cavity, sputum, and ileum were similar, predominantly involving metabolism, environmental and genetic information processing (**Figures 8A–C**).

## Discussion

The oral cavity is the entrance to the digestive tract, which is colonized by one of the most diverse microbiotas of the human body. The human oral microbiome database (HOMD) had recorded about 700 prokaryote species in the human oral cavity, some of which are closely related to human health and disease (Dewhirst et al., 2010). Moreover, growing evidence revealed that some gastrointestinal tract diseases were correlated with dysbiosis of the lung microecology, leading to crosstalk between these organs, termed the gut–lung axis (Budden et al., 2017; Budden et al., 2019). An earlier study showed that more than 48% of adult IBD patients had different degrees of abnormal lung function (Ji et al., 2014), and up to 71% of children and adolescents with CD had abnormal bronchial hyperreactivity (Mansi et al., 2000). Thus, there are emerging evidence that indicated the potential associations between IBD and the lungs (Mateer et al., 2015; Cozzi et al., 2018).

The present study identified the stage-specific alterations of microbiota architecture in the oral cavity, sputum, and ileum of patients with CD. The oral microbiota had a statistically significant decrease in alpha diversity compared to healthy controls, which aligned with Xun’s report (Xun et al., 2018). In addition, we found that the oral microbiota of healthy controls clustered with that from the remission stage of CD, whereas most of the oral microbiota from the active stage of CD clustered separately. A previous study by Zhang et al. (2020) described similar findings. Regarding the composition of oral microbiota of patients with CD, the relative abundances of *Veillonella*, *Actinomyces*, *Megasphaera*, and *Atopobium* were highest in the active stage and lowest in healthy controls. On the contrary, the relative abundances of *Fusobacterium*, *Haemophilus*, and *Porphyromonas* were lowest in the active stage and highest in healthy controls. Said et al. (2014) also reported the depletion of *Haemophilus* in the salivary microbiota of patients with CD. These evidence demonstrated the complicated microbiota signatures in the oral microbiota of patients with CD.

Furthermore, several studies had confirmed the enteric pathogenic potential of *Campylobacter* in the oral cavity (Man, 2011; Ismail et al., 2012; Kirk et al., 2018). Nielsen et al. (2011) showed that oral *Campylobacter* induced impairment of the intestinal barrier function by inducing apoptosis in HT-29/B6 intestinal epithelial cells, providing a plausible explanation to the conclusion reached in this study. Thus, over-growth and colonization of *Campylobacter* in the gastrointestinal tract may contribute to the destruction of cell structures, impairment of the intestinal absorption function, and inflammatory disorders in CD (Qi et al., 2021).

The present study also investigates the characterization of sputum microbiota during the active and remission stages of patients with CD, as this relationship remains understudied. Comparing the structural changes and functional characterization of sputum microbiota of patients with CD, we found that changes in the sputum were similar to those in the oral cavity. Furthermore, the concept of the gut–lung axis is gradually being accepted. The fragments and metabolites derived from intestinal microbiota are transported to the lung and modulated the lung immune response by translocation across the intestinal barrier and the mesenteric lymphatic system (Piersigilli et al., 2020). For instance, intestinal microbiota-mediated production of SCFAs could exert anti-inflammatory effects by modulating immune cell migration and suppressing the activation of nuclear factor kappa B (NF-κB) pathways in the lung (Anand and Mande, 2018). In addition, SCFAs were found to stimulate Treg cells to prevent airway inflammation by inhibiting histone deacetylases (HDACs) or activating acetate and propionate (Thorburn et al., 2015).

Moreover, SCFAs could enhance the generation of dendritic cell precursors, preventing allergic inflammation of the airway (Trompette et al., 2014). Similarly, the dysbiosis of lung microbiota could also affect the gut microbiota (Barcik et al., 2020; Yang et al., 2020). Clinical evidence showed that influenza infection of the lung induced gut microbiota dysbiosis, such as increased *Enterobacteriaceae* and decreased lactococci and lactobacilli, in the intestine (Looft and Allen, 2012). This research may be an opportunity to further explore the relationship between the microbiota in the respiratory tract and CD and provide new options for the clinical treatment of CD.

Until now, the fecal microbiota of patients with CD has been extensively studied. However, the use of fecal samples alone in most of these studies indicates that the findings were specific to the colonic content (Zmora et al., 2018). Recently, Ruigrok et al. (2021) revealed the composition and metabolic potential of the human small intestinal microbiota within the context of IBD. However, no studies revealed the characterization of small intestinal microbiota during different stages of CD, indicating another innovative aspect of our research. Hattori et al. (2020) reported that the abundances of *Streptococcus* and *Clostridium* were higher in the mucosal healing group, which could predict mucosal healing in the small intestine in patients with CD. Our results revealed that these microbiotas were markedly enriched in the remission stage of CD, which further confirmed the above conclusion. Ruigrok et al. (2021) reported *Veillonella*, *Streptococcus*, and *Actinomyces* were abundant in the small intestine of patients with CD. This finding confirmed that the relative abundances of these microbiotas were higher in the remission stage of CD. Several studies had revealed that the colonic microbiota in patients with CD and showed a decrease in probiotics but an increase in the pathogenic microbiota (Dong et al., 2019). Our study demonstrated that the relative abundances of *Lactococcus* and *Lactobacillus* were higher in the remission stage of CD than in the active stage, whereas the contrary was observed for *Fusobacterium*. This suggested that although the structure of the colon was different from that of the small intestine, the abundance of some microbiota may be relatively similar.

While the results of our study offer a detailed insight into the microbiota in multi-ecological sites of patients with CD, some limitations need to be addressed. Since fecal samples from the ileal stoma were collected during the operation, the patients were administrated with antibiotics routinely since the beginning of surgery in the clinic. Currently, we could not estimate the potential influence of antibiotics on the microbiota structure within such a short time, as no strong evidence elucidates these issues so far. Although the influence of antibiotics on results could not be completely avoided, our experimental protocol has minimized the influence. In addition, most previous studies collected samples from healthy controls during routine endoscopies, from patients undergoing intestinal resections or from sudden death individuals (El Aidy et al., 2015). Therefore, it is challenging to collect microbial ileal samples from healthy controls without potential damage. Regarding the collection of sputum samples, the oral cavity is the only way to obtain sputum samples, which inevitably leads to a small amount of oral microbiota mixed in this process. Weiser et al. (2022) revealed that induced sputum captured a microbiota signature representative of the lower airway in 80% of cases and was a direct, non-invasive intervention. Therefore, induced sputum is an essential and practical approach to studying the respiratory microbiota signatures. Perhaps, the contamination can be minimized if a more convenient collection method becomes available, and bioinformatics techniques could also help eliminate the background contamination in the future. In addition, the conclusions of our analyses are also limited by the small sample size. More samples are needed to confirm the changes in some specific microbiota and their roles in the onset of CD.

In conclusion, our study provided insights into the microbiota composition and their functional characterization in the oral cavity, sputum, and ileum of patients with CD compared to healthy controls. Although we only identified the specific microbiota of patients with CD during different disease stages, not referred to the causality, we still displayed the novel microbiota signatures of various sites and their potential relationships with CD. We believe that multi-omics research, including metagenomics, metabolomics, proteomics, and experimental exploration, will provide a more comprehensive understanding of the roles of microbiota in CD, especially on the causality between microbiota and CD, which will improve the treatment strategy for CD in the future.

## Data Availability Statement

The datasets presented in this study can be found in online repositories. The names of the repository/repositories and accession number(s) can be found below: https://www.ncbi.nlm.nih.gov/, PRJNA773617.

## Ethics Statement

The studies involving human participants were reviewed and approved by the Ethics Committee of Shanghai Tenth People’s Hospital. The patients/participants provided their written informed consent to participate in this study. Written informed consent was not obtained from the individual(s) for the publication of any potentially identifiable images or data included in this article.

## Author Contributions

KX experimental design, implementation research, analysis and interpretation of data, and drafting of papers. RG experimental design, implementation research, analysis and interpretation of data, and revision of papers. XW, JS, JW, and TW experimental design, analysis and interpretation of data, and revision of papers. JF, LY, and CC experimental design, revision of papers, and funding support. All authors contributed to the article and approved the submitted version.

## Conflict of Interest

The authors declare that the research was conducted in the absence of any commercial or financial relationships that could be construed as a potential conflict of interest.

## Publisher’s Note

All claims expressed in this article are solely those of the authors and do not necessarily represent those of their affiliated organizations, or those of the publisher, the editors and the reviewers. Any product that may be evaluated in this article, or claim that may be made by its manufacturer, is not guaranteed or endorsed by the publisher.

## Supplementary Material

Supplementary Figure 1 Structure analysis of the ileal microbiota in patients with CD. **(A, B)** Chao diversity index of phylum level and genus level in the ileal samples. **(C, D)** Shannon diversity index of phylum level and genus level in the ileal samples. **(E, F)** Venn diagram indicating the overlap of OTUs in the categories on the phylum level and genus level, respectively.

Supplementary Figure 2 COG functional classifications of microbiota in patients with CD. **(A–C)** COG functional classifications of microbiota in the oral cavity, sputum and ileum, respectively.
