# Supplementary figures and images for: Characterization of Specific Signatures of the Oral Cavity, Sputum, and Ileum Microbiota in Patients With Crohn’s Disease
Source: Front Cell Infect Microbiol. 2022 Apr 13;12:864944. doi: 10.3389/fcimb.2022.864944 (PMC9045729; doi:10.3389/fcimb.2022.864944)

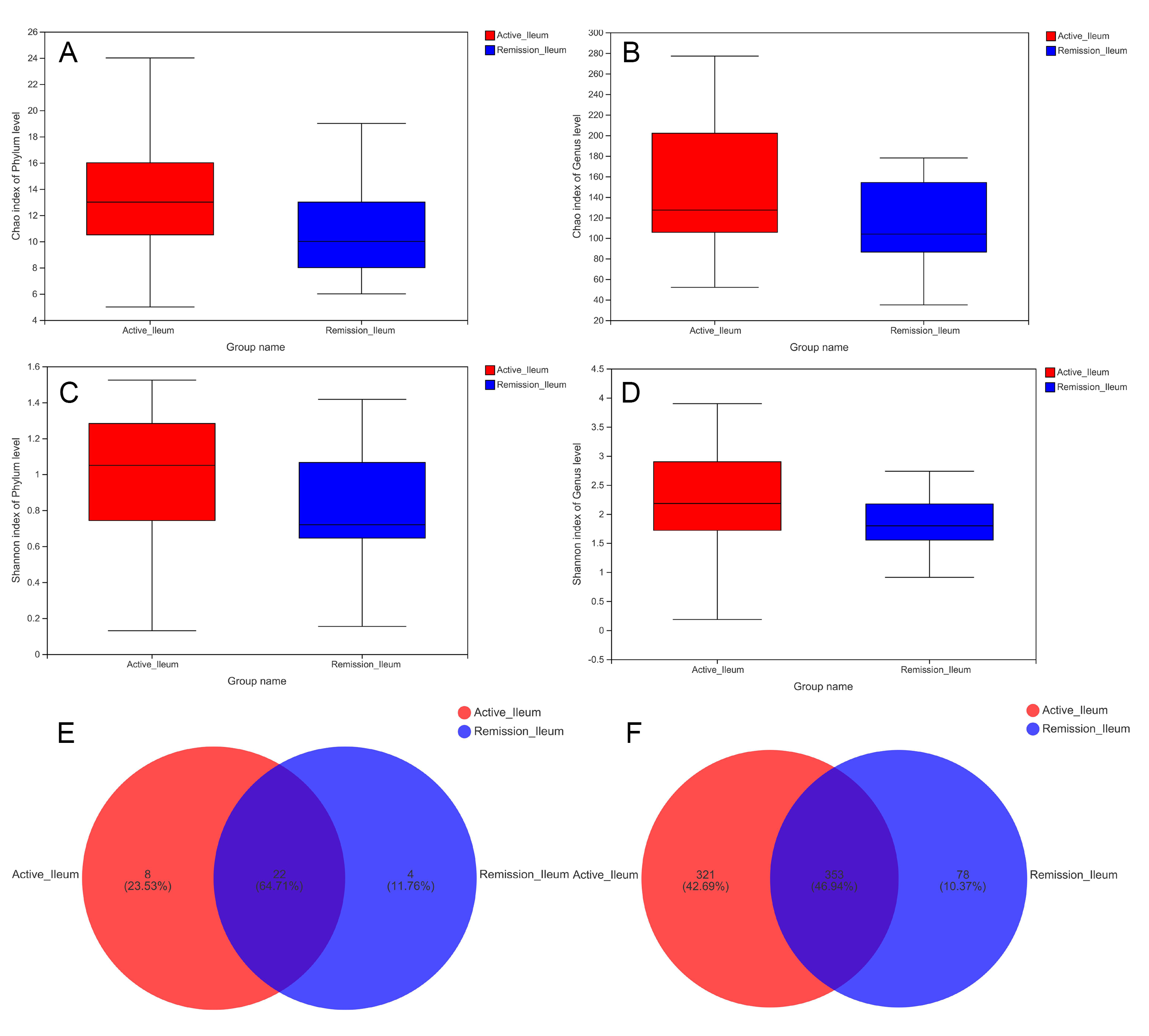

Supplement: Supplementary Figure 1 — Structure analysis of the ileal microbiota in patients with CD. (A, B) Chao diversity index of phylum level and genus level in the ileal samples. (C, D) Shannon diversity index of phylum level and genus level in the ileal samples. (E, F) Venn diagram indicating the overlap of OTUs in the categories on the phylum level and genus level, respectively. [file Image_1.tif]

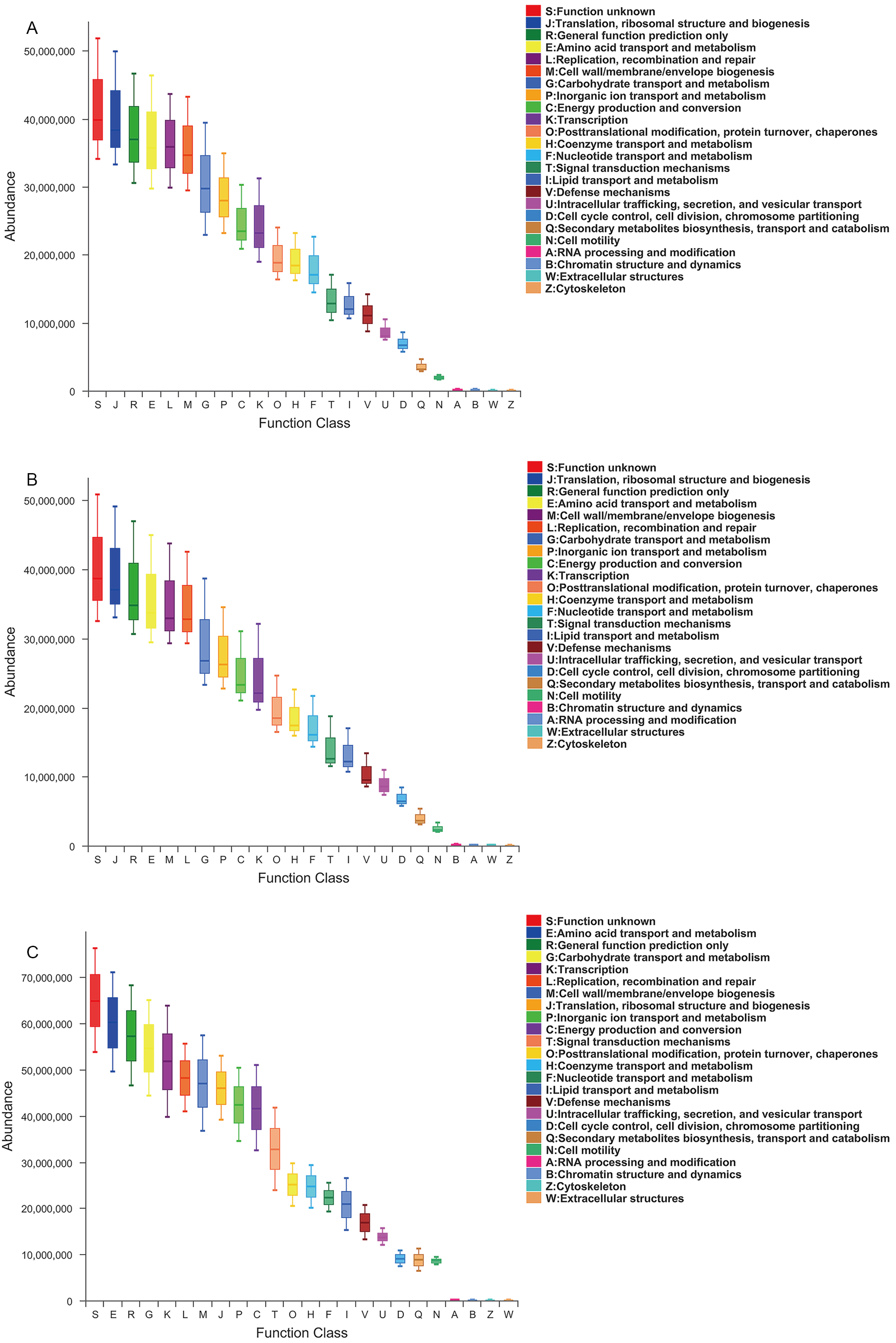

Supplement: Supplementary Figure 2 — COG functional classifications of microbiota in patients with CD. (A–C) COG functional classifications of microbiota in the oral cavity, sputum and ileum, respectively. [file Image_2.tif]
